# Anti-Inflammatory Effects of Rebamipide Eyedrop Administration on Ocular Lesions in a Murine Model of Primary Sjögren's Syndrome

**DOI:** 10.1371/journal.pone.0098390

**Published:** 2014-05-27

**Authors:** Rieko Arakaki, Hiroshi Eguchi, Akiko Yamada, Yasusei Kudo, Akihiko Iwasa, Tserennadmid Enkhmaa, Fumika Hotta, Sayaka Mitamura-Aizawa, Yoshinori Mitamura, Yoshio Hayashi, Naozumi Ishimaru

**Affiliations:** 1 Department of Oral Molecular Pathology, Institute of Health Biosciences, The University of Tokushima Graduate School, Tokushima, Japan; 2 Department of Ophthalmology, Institute of Health Biosciences, The University of Tokushima Graduate School, Tokushima, Japan; National Institute of Dental and Craniofacial Research, United States of America

## Abstract

**Background:**

Topical therapy is effective for dry eye, and its prolonged effects should help in maintaining the quality of life of patients with dry eye. We previously reported that the oral administration of rebamipide (Reb), a mucosal protective agent, had a potent therapeutic effect on autoimmune lesions in a murine model of Sjögren's syndrome (SS). However, the effects of topical treatment with Reb eyedrops on the ocular lesions in the murine model of SS are unknown.

**Methods and Finding:**

Reb eyedrops were administered to the murine model of SS aged 4–8 weeks four times daily. Inflammatory lesions of the extraorbital and intraorbital lacrimal glands and Harderian gland tissues were histologically evaluated. The direct effects of Reb on the lacrimal glands were analyzed using cultured lacrimal gland cells. Tear secretions of Reb-treated mice were significantly increased compared with those of untreated mice. In addition to the therapeutic effect of Reb treatment on keratoconjunctivitis, severe inflammatory lesions of intraorbital lacrimal gland tissues in this model of SS were resolved. The mRNA expression levels of IL-10 and mucin 5Ac in conjunctival tissues from Reb-treated mice was significantly increased compared with those of control mice. Moreover, lactoferrin production from lacrimal gland cells was restored by Reb treatment.

**Conclusion:**

Topical Reb administration had an anti-inflammatory effect on the ocular autoimmune lesions in the murine model of SS and a protective effect on the ocular surfaces.

## Introduction

Sjögren's syndrome (SS) is an autoimmune disease characterized by lymphocyte infiltration and destruction of the salivary and lacrimal glands [1]. Dry eye and dry mouth are the main clinical manifestations of SS. In particular, many patients with SS suffer from keratoconjunctivitis due to severe dry eye. The typical symptoms of keratoconjunctivitis sicca include burning, itchiness, pain in the eyes, or tired eyes [2], [3]. The quality of life of patients with SS is significantly impaired; therefore, an effective therapy for ocular lesions would improve the quality of life of these patients.

In a previous report, we demonstrated the therapeutic effects of orally administered rebamipide (Reb), a mucosal protective agent, on autoimmune lesions in thymectomized NFS/*sld* mice, a murine model for SS [4]. Reb had two potent effects including the immunosuppressive activity and the antiapoptotic activity in target cells [4]. In addition, recent studies have shown that Reb increased the barrier function of the intestinal mucosa, gastric mucosa, and corneal epithelial cells [5]–[8]. However, the possible effects of Reb on ocular lesions in an animal model of SS have not been determined.

Various tissues in the orbit comprise the eyeball, fat tissue, muscles, connective tissue, and lacrimal glands [9]. Although evidence indicates that ocular lesions in SS involve injury to the epithelial cells of the conjunctiva and cornea, the sources of inflammation of the lacrimal glands or accessory glands in the orbit remain unclear.

In the present study, treatment with Reb eyedrops was administered to our model of primary SS [10] to determine the therapeutic effects of Reb on ocular inflammatory lesions in SS. Our findings could be important for indicating an effective topical medication for patients with SS.

## Results

### The effects of Reb eyedrops on tear secretion in the murine model of SS

**Figure 1:**
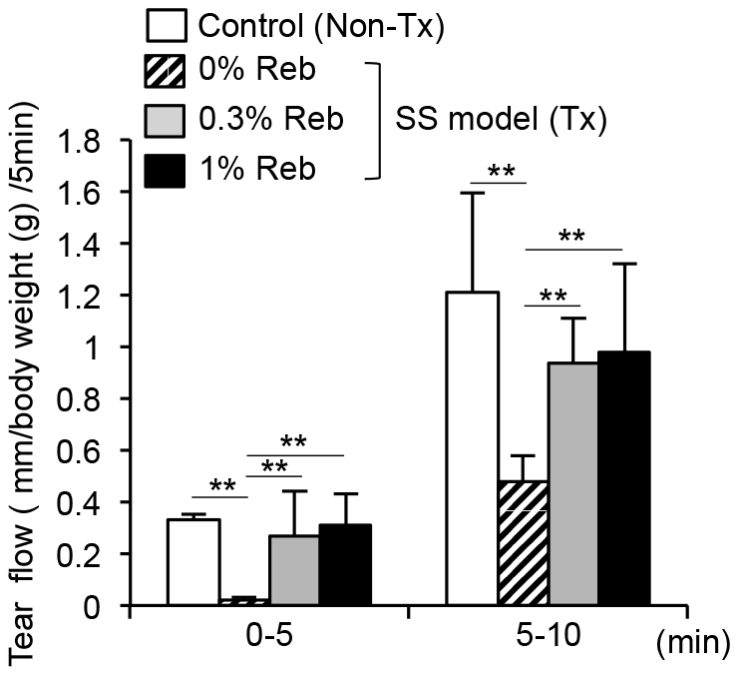
Therapeutic effects of Reb eyedrop administration on tear secretion in the murine model of SS. Average tear volumes after pilocarpine administration (2.5 mg/kg) were measured for 5 min (0–5 min and 5–10 min). Results are expressed as means ± SD for 5 mice per group. Control, nonthymectomized (non-Tx) NFS/*sld* mice. ** *P*<0.005**.**

NFS/*sld* mice were thymectomized 3 days (3d-Tx) after birth, and 3d-Tx female mice aged 4 weeks were used for experiments. Reb eyedrops (0%, 0.3%, and 1%) were administered four times daily to mice aged 4–8 weeks. Tear volumes were measured to determine the effects of Reb treatment on tear fluid secretion. The average decreased tear volumes in 0% Reb-treated mice were restored by treatment with 0.3% and 1% Reb eyedrops and were similar to tear volumes of untreated non-Tx mice (**Figure 1**).

### The effects of Reb eyedrops on ocular lesions in the murine model of SS

**Figure 2:**
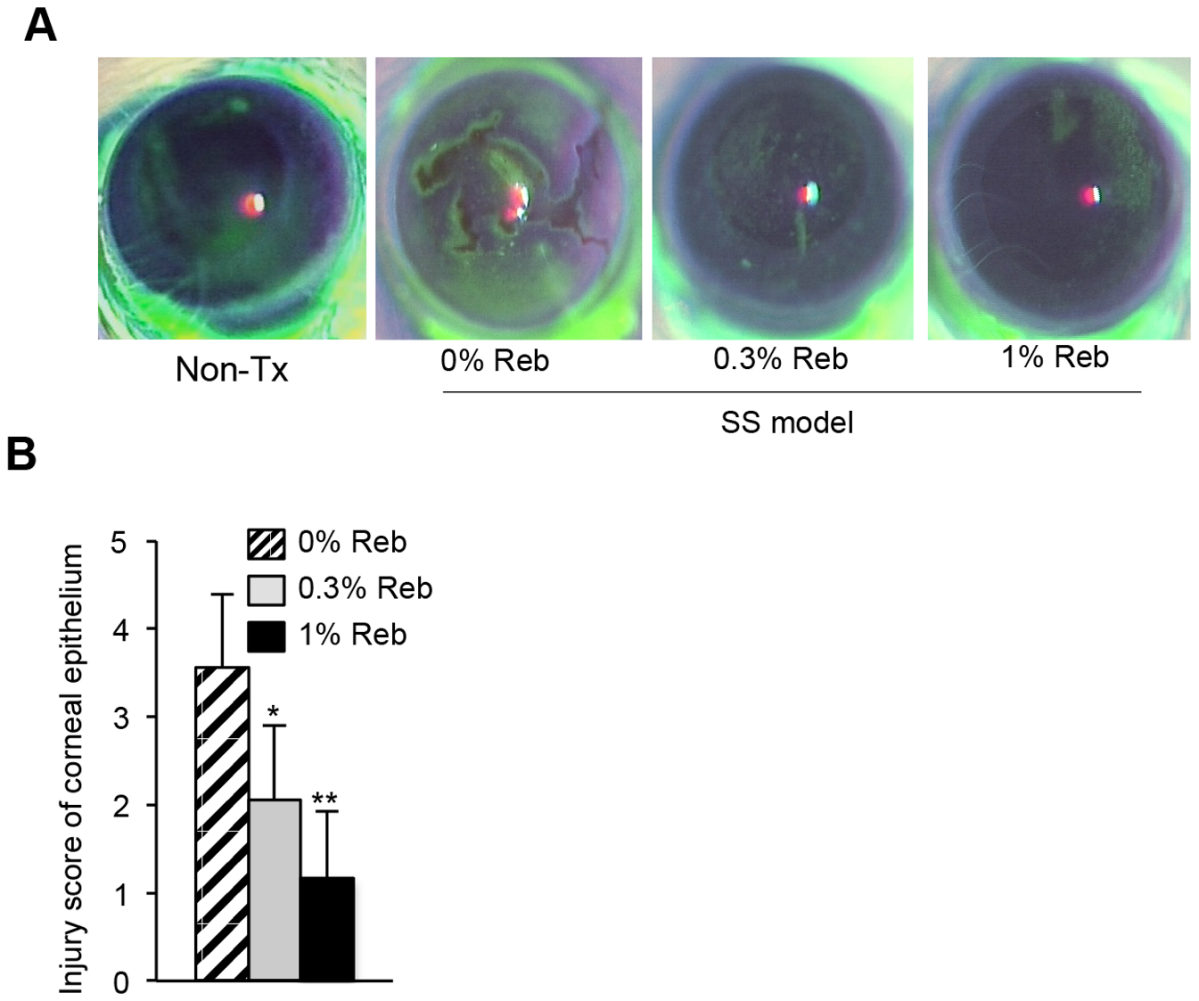
Effect of Reb eyedrops on the ocular lesions in the murine model of SS. (A) Ocular surface lesions in Reb-treated mice were examined using fluorescein staining of the ocular surface. Representative photomicrographs for each group are shown. (B) The scores for corneal epithelial disorders. Results are expressed as mean ± SD for five mice per group. *P<0.05, **P<0.005.

Mice were assessed for corneal epithelial disorders using fluorescein staining. The corneal epithelium of 3d-Tx mice showed sparse or dense superficial punctate keratitis similar to that of patients with SS, whereas the corneal epithelium of non-Tx mice were not detectably stained (**Figure 2A**). The corneal epithelium of Reb-treated (0.3% and 1%) mice was improved (**Figure 2A**). The fluorescein staining scores of Reb-treated 3d-Tx mice were significantly lower compared with of control mice in a dose-dependent manner (**Figure 2B**).

### Histopathological changes in inflammatory lesions of lacrimal gland tissues after treatment with Reb eyedrops

**Figure 3:**
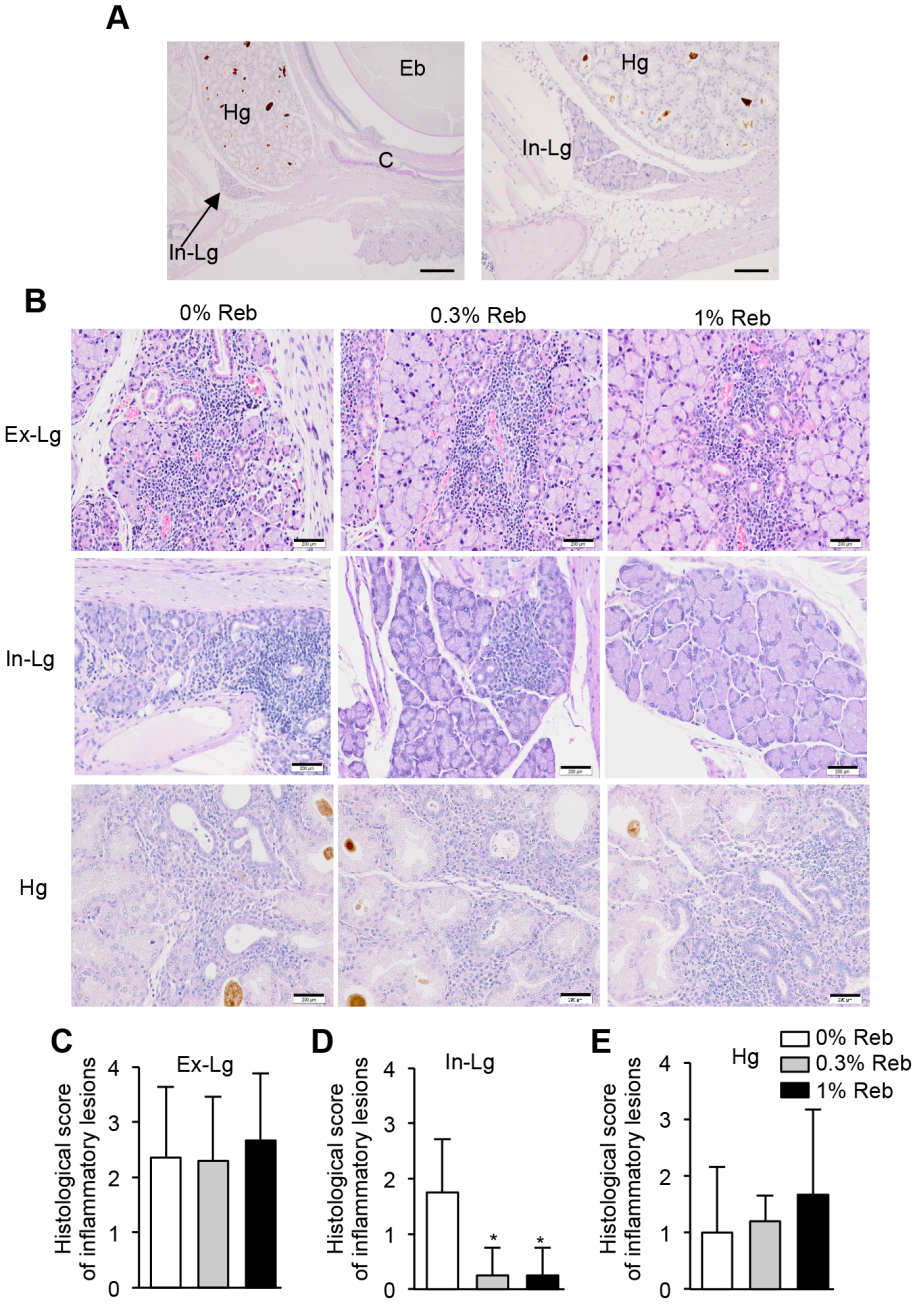
Inflammatory lesions of lacrimal glands and Harderian glands (Hg) in Reb-treated murine model of SS. (A) Anatomical positions of the intraorbital lacrimal glands (In-Lg) and Hg of normal mice. C, cornea; Eb, eyeball. Scale bar  =  200 µm (left), 100 µm (right). (B) Inflammatory lesions in the extraorbital lacrimal glands (Ex-Lg), In-Lg, and Hg of Reb-treated murine model of SS. Photomicrographs are representative of five mice per group. Scale bars  =  200 µm. (C, D, E) Histological scores of inflammatory lesions in the Ex-Lg (C), In-Lg (D), and Hg (E). Results expressed as mean ± SD for five mice per group. *P<0.05.

The lacrimal glands include the extraorbital and intraorbital glands. We previously reported that inflammatory lesions of the salivary glands, but not those of the extraorbital lacrimal glands (Ex-Lg), resolved after the oral administration of Reb [4]. In addition to the Ex-Lg, the intraorbital lacrimal glands (In-Lg) and Harderian glands (Hg) are known to maintain ocular mucosal tissues [9]. In-Lg and Hg tissues of untreated non-Tx mice are shown in **Figure 3A**. In this model of SS, inflammatory lesions were detected in the Ex-Lg, In-Lg, and Hg (**Figure 3B**). Mononuclear lymphocytes are infiltrated around the duct or adjacent the gland cells in lacrimal gland tissues of 3d-Tx mice (**Figure 3B**). No changes were observed in inflammatory lesions of the Ex-Lg and Hg of Reb-treated mice (**Figure 3B** **)**. In contrast, inflammatory lesions in the In-Lg of Reb-treated mice improved (**Figure 3B**). Moreover, the histological scores for the In-Lg, but not Ex-Lg, of 0.3% and 1% Reb-treated mice were significantly lower compared with those of 0% Reb-treated control mice (**Figure 3C, D**). The scores for Hg were not changed by Reb eyedrop treatment (**Figure 3E**).

### The anti-inflammatory effects of Reb on the ocular lesions in the murine model of SS

**Figure 4:**
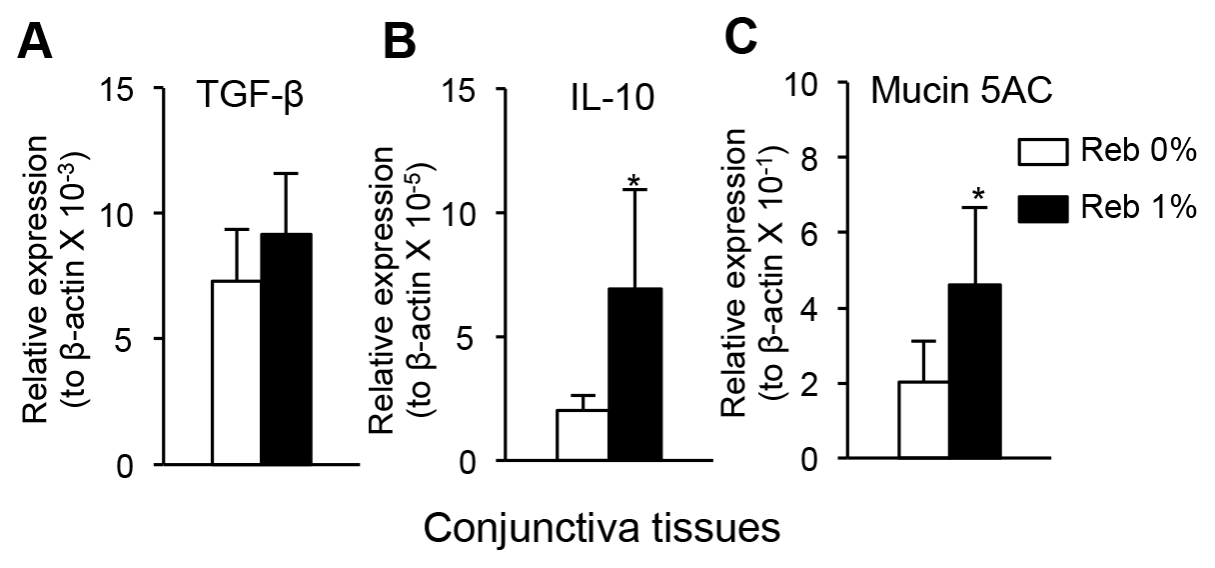
Anti-inflammatory proteins in the ocular lesions in Reb-treated murine model of SS. The mRNA expression levels of TGF-β (A), IL-10 (B), and mucin 5AC (C) in conjunctival tissues in Reb-treated (0% and 1%) mice were analyzed using real-time RT-PCR. Results expressed as mean ± SD for five mice per group.

To determine the molecular mechanisms underlying the therapeutic effects of Reb eyedrop treatment, the mRNA expression levels of immunoregulatory cytokines and protective proteins [11]–[13], including IL-10, TGF-β, and mucin 5AC, in conjunctival tissues were analyzed using real-time RT-PCR (**Figure 4A, B, and C**). The mRNA expression levels of IL-10 and mucin 5AC in Reb-treated mice were significantly increased compared with those in Reb 0%-treated mice (**Figure 4B, C**).

### The effect of Reb on lactoferrin secretion by lacrimal glands in the murine model of SS

**Figure 5:**
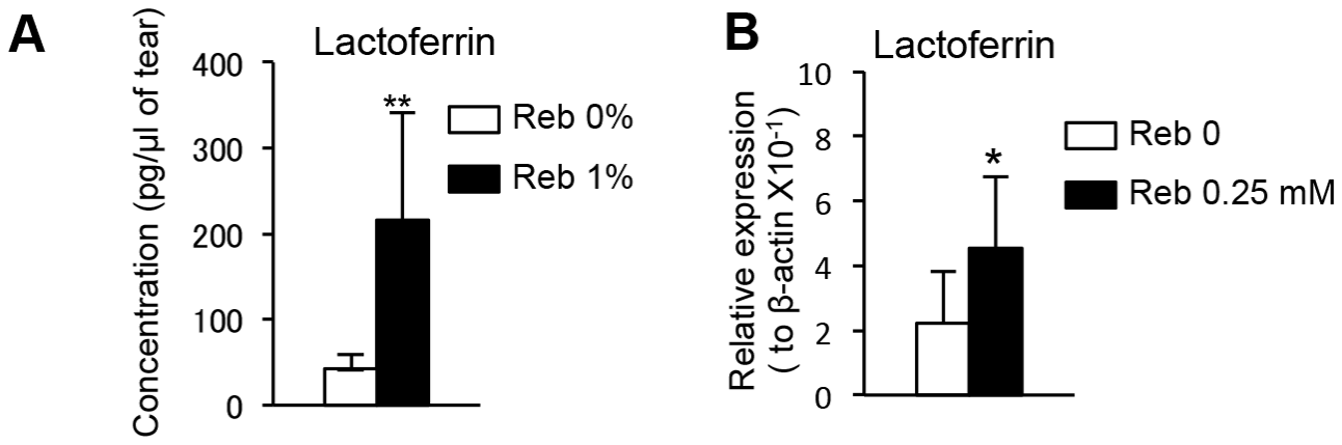
Lactoferrin in tears from Reb-treated mice and direct effect of Reb on lacrimal glands. (A) The lactoferrin concentrations in the tears from Reb-treated mice were determined using ELISA. Results are expressed as mean ± SD for five mice per group. (B) Lacrimal gland cells from Tx-NFS/*sld* mice were cultured with or without Reb (0.25 mM) for 6 h, and the lactoferrin mRNA levels in cultured Lg fragments were then analyzed using real-time RT-PCR. Results are expressed as mean ± SD of triplicate determinations per group from 3 independent experiments. *P<0.05, **P<0.01.

The results of ELISA showed that the concentration of the anti-inflammatory factor lactoferrin [14]–[17] in the tears of Reb-treated mice was significantly increased compared with that of 0% Reb-treated mice (**Figure 5A**). In the analysis of the mRNA expressions levels of lactoferrin in cultured tissues using real-time RT-PCR, the mRNA expression levels of lactoferrin in cultured lacrimal gland tissues were more promoted by addition of Reb (**Figure 5B**).

### Increased expression of lactoferrin in In-Lg by Reb eyedrop therapy

**Figure 6:**
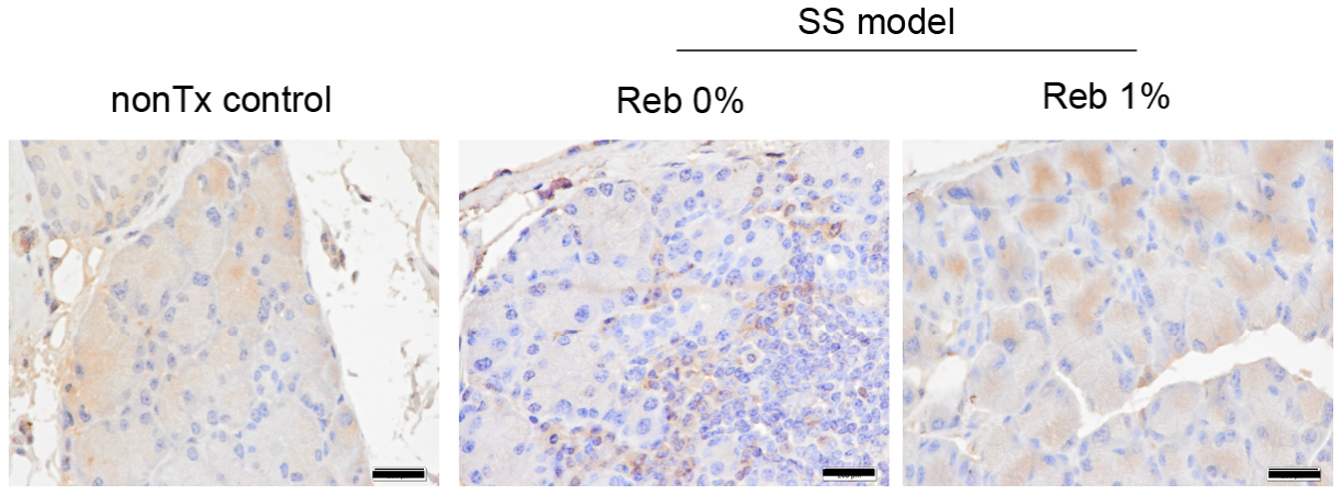
Change of lactoferrin expression by Reb eyedrop treatment. Lactoferrin expression in In-Lg tissues of non-Tx control mice, 0%Reb-treated model of SS, and 1% Reb-treated murine model of SS was detected using immunohistochemistry. Images are representative of five mice per each group. Scale bar  =  100 µm.

Using immunohistochemistry, we found that the expression of lactoferrin in In-Lg tissues of the murine model of SS was significantly reduced compared with that in of normal In-Lg tissues (**Figure 6**). Further, immune cells such as macrophages were positive for lactoferrin (**Figure 6**). Treatment with Reb restored lactoferrin expression in In-Lg tissues of the murine model of SS (**Figure 6**).

## Discussion

Reb has been widely applied as a gastroprotective drug against gastritis and gastric ulcers, and it exhibits mucin secretagogue activity, anti-inflammatory actions, and antibacterial effects [6], [7], [18], [19]. In a previous report, we demonstrated that the oral administration of Reb had a therapeutic effect on autoimmune lesions of the salivary glands in thymectomized NFS/*sld* mice, a murine model of SS [4]. In that paper, Reb treatment inhibited the activation of effector T cells, and the production of Th1-type cytokines such as IL-2 and IFN-γ and was associated with decreased NF-κB activity [4]. Moreover, the serum autoantibody levels were clearly decreased after the oral administration of Reb [4]. Although oral administration of Reb at a low concentration resolved the inflammatory lesions of the salivary glands in our murine model of SS and restored saliva secretion, inflammatory lesions of the lacrimal glands were not resolved by the oral administration of Reb at the same dose [4]. Moreover, tear secretion in this model of SS was not recovered after oral administration [4]. On the other hand, Kinoshita et al. demonstrate that 2% rebamipide ophthalmic suspension is effective in improving both the objective signs and subjective symptoms of dry eye patients including primary or secondary SS patients (16.9%) [20]. Our findings as for the objective signs including fluorescein corneal staining score and pathological score of intraorbital lacrimal gland in rebamipide-treated SS model mice are similar to those in dry eye patients. We believe that our results show the anti-inflammatory effects of rebamipide on the ocular lesions in murine suggest the same effects on human ocular surface, and the effect contribute improving subjective symptoms. Our results would be useful for establishing more effective therapeutic strategy of SS with rebamipide.

In our murine model of SS, neonatal thymectomy was performed to induce the breakdown of central or peripheral tolerance to trigger autoimmunity [10], [21]. The thymic function of almost all patients with SS may be intact. Although MRL/*lpr* or NZB/NZW F1 mice are also well known to be an animal model of SS, they are considered as a model of secondary SS with systemic autoimmune lesions [22]–[24]. In contrast, thymectomized NFS/*sld* mice have been established as a model of primary SS [10]. As inflammatory lesions in this model are localized in salivary and lacrimal glands, resembling human primary SS, and immune phenotypes and responses including cytokine profile, autoantibody production, or antigen-specific T cell responses are similar to those of human SS, this animal model has been used for analyzing the pathogenesis of SS and establishing new therapeutic strategies [25]–[27]. In addition, autoimmune lesions have an earlier onset in young mice (aged 6–8 weeks), with the frequency of onset being almost 100%. Therefore, this model is considerably useful for the study of SS despite several large differences between humans and mice. On the other hand, there are no differences in body weight, gross pelage aspect, reproductive capacity between control and thymectomized NFS/*sld* mice. As for immunological aspects such as decreased T cell number, enhanced Th1-type cytokine production, and autoantibody production in addition to autoimmune lesions in salivary and lacrimal glands were observed in the thymectomized mice [25]–[27].

With respect to the endocrine system, female-specific autoimmune lesions were observed in thymectomized NFS/*sld* mice, resembling human SS [10], [21]. Moreover, autoimmune lesions in the murine model of SS that underwent ovariectomy were markedly enhanced compared with those in sham-operated mice, suggesting that sex hormones such as estrogen play a key role in the onset of autoimmune lesions in SS [28]. Although the relationship between thymectomy and endocrine system in this model is still unclear, some interaction may occur between the immune and endocrine systems to trigger the onset of SS.

In the present study, we show that Reb eyedrops had therapeutic effects on ocular lesions and tear secretion in thymectomized NFS/*sld* mice. Thus, inflammatory lesions of the In-Lg, but not Ex-Lg, in the murine model had clearly improved after Reb eyedrop treatment. This finding suggests that Reb exerts a potent anti-inflammatory action against an autoimmune response in the exocrine glands, and it is also exerts protective effects on the ocular surface. Because of the anatomical structure of the orbit, drugs are unable to reach the Ex-Lg. Considering the therapeutic effect of Reb eyedrops on the In-Lg, it is possible that In-Lg plays an important role in maintaining ocular and orbital tissues.

Ex-Lg tissues were analyzed in studies of most animal models of SS [29]. Because the systemic exocrine glands are targeted in SS, In-Lg tissues are also targets. Similarly, the minor salivary glands in the lip, oral palate, or oral mucosa are known to be targeted in SS as well as the submandibular, sublingual, and parotid glands, which are the major salivary glands [30]. The Hg and In-Lg are present in mice, but not in humans, and act as accessories to the lacrimal glands by secreting mucus or lipids [31], while the accessory lacrimal glands are present in the conjunctival tissue of humans. Because Reb eyedrops exerted no effects on these inflammatory lesions in the Ex-Lg and Hg, anatomical or functional differences between the lacrimal glands may influence their efficiency. Much of the tear volume seems to be secreted by the Ex-Lg and Hg. However, the In-Lg may supply tears for dry eye due to the dysfunction of the major lacrimal glands to maintain the ocular surface. It is possible that the In-Lg may play a key role in the speedy recovery from dryness of the ocular surface. Hg in mice is known to play an important role in maintenance of ocular functions, and it was reported that pilocarpine can stimulate significant tear secretion from Harderian gland in mice [32], [33]. Although inflammatory lesions of Hg in Reb-treated SS model was not recovered, any protective effect of Reb may influence the gland to promote tear secretion. We believe that rebamipide may act as both an anti-inflammatory and mucosa-protective agent.

The mRNA expression levels of several key factors that mediate conjunctiva tissue function, including IL-10 and mucin 5AC, were changed by Reb treatment. Further, our findings suggest that the effect of Reb through the upregulation of lactoferrin in the lacrimal glands may play a key immunoregulatory role in autoimmune responses. It was reported that lactoferrin targets T cells to maintain mucosal immunity [34]. Moreover, it was demonstrated that lactoferrin prevents lacrimal gland dysfunction in older mice [35]. A recent report suggested that Reb increased the barrier function and controlled TNF-α-induced barrier disruption via modulating cytokine expression in corneal epithelial cells [7]. Reb may contribute to the anti-inflammatory effect on exocrine glands or mucosal membrane through cytokines or growth factors.

Most opthalmologists generally agree with the fact that topically applied eyedrops penetrate into adjacent tissues mainly through the cornea and partially through the conjunctiva. Many cases of vernal keratoconjunctivitis with giant papillary conjunctivitis can be treated using the topical administration of steroids or immunosuppressants, and the giant papillae reduce in size. An epidemiological study accounts for the fact that topically applied eyedrops partially penetrate into the conjunctiva [36]. We believe that it would be reasonable to conclude that rebamipide partially penetrates into the conjunctival papillae and works as an anti-inflammatory agent because the In-Lg is located close to the conjunctiva papillae.

In conclusion, we showed here that Reb exerts both anti-inflammatory and mucosal protective functions for effective treatment of ocular lesions that occur in SS. Because the molecular mechanisms underlying the therapeutic effects of topical Reb administration remain unclear, further research is required to establish appropriate treatments for SS.

## Materials and Methods

### Ethics

This study was conducted according to the Fundamental Guidelines for Proper conduct of Animal Experiment and related Activities in Academic Research Institutions under the jurisdiction of the Ministry of Education, Culture, Sports, Science and Technology of Japanese Government. The protocol was approved by the Committee on the Animal Experiments of the University of Tokushima (Permit Number: toku-09021). All experiment was performed under ketamine and xylazin anesthesia, and all efforts were made to minimize suffering.

### Mice and experimental design

NFS/*sld* female mice with a mutant *sld* were reared in our specific pathogen-free mouse colony and were provided with food and water ad libitum at a constant ambient temperature (22−24°C) under a constant day-night rhythm. Thymectomy was performed on these mice on day 3 after birth [10]. Reb eyedrops (0%, 0.3%, and 1%) were formulated at Otsuka Pharmaceutical Co. Ltd., and 2 µl of these drops was applied to each eye of mice aged 4–8 weeks four times daily. After the administration during 4 weeks, mice were analyzed.

### Tear secretion measurements

Tear volumes of Reb-treated mice were determined using a previously described method [37], [38]. In brief, to measure tear secretion in pilocarpine-stimulated mice, mice were anesthetized with ketamine (60 mg/kg body weight) and xylazin (6 mg/kg), and then intraperitoneally injected with pilocarpine (2.5 mg/kg) (Wako, Osaka, Japan). Tear volume was determined by measuring the length of the phenol-red thread (Showa Yakuhin Kako Co., Ltd., Tokyo, Japan) left in contact with the eye every 5 min after injection of pilocarpine until 10 min. Tear secretion was calculated as tear volume/body weight.

### Evaluation of ocular surfaces

We prepared a fluorescein liquid by dilating Fluorescite intravenous injection 500 mg (Alcon Japan, Tokyo) by 2% with saline. One to 2 µL of the liquid was administered to the cornea by micropipette, and the residual liquids around the eye were washed by saline and wiped by filter paper gently. After fluorescein staining of the eye, the cornea was examined for corneal epithelial disorders using a slit-lamp microscope with a blue filter; these disorders were evaluated using a modified, previously described method [39]. In brief, corneal epithelial disorders were scored on a scale of 0–3 as follows: 0, no punctate staining (normal); 1, sparse punctate staining; 2, intermediate staining between 1 and 3; and 3, dense punctate staining. The analysis was performed in a double-blind manner.

### Histological evaluations

After the mice were sacrificed, all the organs were removed, fixed in 10% phosphate-buffered formaldehyde (pH 7.2), and prepared for histological examination. Formalin-fixed tissue sections were stained with hematoxylin and eosin. These were independently evaluated by three pathologists in a blinded manner. Inflammatory lesions of the lacrimal glands were histopathologically evaluated as previously described [40].

### Quantitative real-time reverse transcription-polymerase chain reaction analysis

Total RNA was extracted from conjunctival tissues using ISOGEN (Wako Pure Chemical Industries, Ltd., Osaka, Japan) was and reverse transcribed. Transcripts of target genes and β-actin were prepared using a 7300 Real-Time PCR System (Applied Biosystems, Foster City, CA) with SYBR Premix Ex Taq (Takara Bio, Shiga, Japan). The primer sequences used were as follows: TGF-β: forward, 5′-GACCGCAACAACGCCATCTAT-3′ and reverse, 5′-GGCGTATCAGTGGGGGTCAG-3′; IL-10: forward, 5′-ATCGATTTCTCCCCTGTGAA-3′ and reverse, 5′-TGTCAAATTCATTCATGGCCT-3′; mucin 5AC: forward, 5′-AAAGACACCAGTAGTCACTCAGCAA-3′ and reverse, 5′-CTGGGAAGTCAGTGTCAAACCA-3′; lactoferrin: forward, 5′-ACAATGCTGGAGATGTGGCT-3′ and reverse, 5′-TTGTCATTCGTGCTTCGGGA-3′; and β-actin: forward, 5′-GTGGGCCGCTCTAGGCACCA-3′ and reverse, 5′-CGGTTGGCCTTAGGGTTCAGGGGG-3′. Relative mRNA abundance of each transcript was normalized against β-actin.

### Lactoferrin in tears

In mice injected with pilocarpine (2.5 mg/kg), tears from the lateral canthus were collected using a 5-µl glass capillary tube (Hirschmann Laborgeräte GmbH & Co., Eberstadt, Germany) for 20 min. Collected tears were suspended in Pro-prep protein extraction solution (iNtRON Biotechnology, Kyunggi-do, Korea) and centrifuged, and supernatants were added to phosphate-buffered saline (final volume: 100 µl). The concentrations of lactoferrin in tears were determined using a Lactoferrin ELISA kit (USCN Life Science Inc., Wuhan, China).

### Organ culture of extraorbital lacrimal glands (Ex-Lg)

Female thymectomized NFS/*sld* mice were anesthetized, and the lacrimal gland tissues were resected. The Ex-Lg were cut into pieces less than 1 mm^3^. Tissue fragments were cultured at 37°C with or without 0.25 mM Reb in a highly gas-permeable Imaging Plate FC (Zell-Kontakt GmbH, Nörten-Hardenberg, Germany) containing Dulbecco's modified Eagle medium (DMEM) with 10% fetal bovine serum, 2.5 µM dexamethasone, 10 µg/ml insulin, 5.5 µg/ml transferrin, 6.7 ng/ml sodium selenite, 25 µg/ml ascorbic acid, 10 ng/ml epidermal growth factor, 10 µg/ml reduced glutathione, 5 µg/ml amphotericin B, 100 µg/ml streptomycin, and 100 units/ml penicillin in an atmosphere containing 5% CO_2_. After culture for 6 h, total RNA was prepared from these cultured fragments for quantitative RT-PCR analysis to determine the direct effects of Reb on exocrine gland tissues.

### Immunohistochemistry

Paraffin-embedded sections were stained with rabbit anti-lactoferrin antibodies (Abcam) using the biotin-avidin immunoperoxidase complex reagent (Dako). Nuclei were counterstained with hematoxylin.

### Statistical analysis

Statistical comparisons were performed using the Dunnett's (**Figure 1**), lower-tailed Shirley–Williams (**Figures 2** **and** **3**), and unpaired Student *t*-tests (**Figures** **4** **and** **5**).
